# Mechanistic inhibition of Monkeypox and Marburg virus infection by O-rhamnosides and Kaempferol-o-rhamnosides derivatives: a new-fangled computational approach

**DOI:** 10.3389/fcimb.2023.1188763

**Published:** 2023-05-24

**Authors:** Md. Abdullah Al Mashud, Ajoy Kumer, Nobendu Mukerjee, Akhel Chandro, Swastika Maitra, Unesco Chakma, Abhijit Dey, Shopnil Akash, Athanasiosis Alexiou, Azmat Ali Khan, Amer M. Alanazi, Arabinda Ghosh, Kow-Tong Chen, Rohit Sharma

**Affiliations:** ^1^ Biophysics and Biomedicine Research Lab, Department of Electrical & Electronic Engineering, Islamic University, Kushtia, Bangladesh; ^2^ Laboratory of Computational Research for Drug Design and Material Science, Department of Chemistry, European University of Bangladesh, Dhaka, Bangladesh; ^3^ Department of Microbiology, West Bengal State University, West Bengal, Kolkata, India; ^4^ Department of Health Sciences, Novel Global Community Educational Foundation, Habersham, NSW, Australia; ^5^ Department of Poultry Science, Faculty of Animal Science & Veterinary Medicine, Sher-e-Bangla Agricultural University, Dhaka, Bangladesh; ^6^ Department of Microbiology, Adamas University, West Bengal, Kolkata, India; ^7^ School of Electronic Science and Engineering, Southeast University, Nanjing, China; ^8^ Department of Life Sciences, Presidency University, Kolkata, West Bengal, India; ^9^ Department of Pharmacy, Daffodil International University, Sukrabad, Dhaka, Bangladesh; ^10^ Department of Science and Engineering, Novel Global Community Educational Foundation, Habersham, NSW, Australia; ^11^ Department of Neuroscience, AFNP Med, Wien, Austria; ^12^ Pharmaceutical Biotechnology Laboratory, Department of Pharmaceutical Chemistry, College of Pharmacy, King Saud University, Riyadh, Saudi Arabia; ^13^ Microbiology Division, Department of Botany, Gauhati University, Assam, India; ^14^ Department of Occupational Medicine, Tainan Municipal Hospital (managed by Show Chwan Medical Care Corporation), Tainan, Taiwan; ^15^ Department of Public Health, College of Medicine, National Cheng Kung University, Tainan, Taiwan; ^16^ Department of Rasa Shastra and Bhaishajya Kalpana, Faculty of Ayurveda, Institute of Medical Sciences, Banaras Hindu University, Varanasi, Uttar Pradesh, India

**Keywords:** Monkeypox virus, Marburg virus, drug development, O-rhamnosides, Kaempferol-o-rhamnosides, admet, molecular docking, molecular dynamics simulations

## Abstract

The increasing incidence of Monkeypox virus (Mpox) and Marburg virus (MARV) infections worldwide presents a significant challenge to global health, as limited treatment options are currently available. This study investigates the potential of several O-rhamnosides and Kaempferol-O-rhamnosides as Mpox and MARV inhibitors using molecular modeling methods, including ADMET, molecular docking, and molecular dynamics/MD simulation. The effectiveness of these compounds against the viruses was assessed using the Prediction of Activity Spectra for Substances (PASS) prediction. The study’s primary focus is molecular docking prediction, which demonstrated that ligands (L07, L08, and L09) bind to Mpox (PDB ID: 4QWO) and MARV (PDB ID: 4OR8) with binding affinities ranging from -8.00 kcal/mol to -9.5 kcal/mol. HOMO-LUMO based quantum calculations were employed to determine the HOMO-LUMO gap of frontier molecular orbitals (FMOs) and to estimate chemical potential, electronegativity, hardness, and softness. Drug similarity and ADMET prediction assessments of pharmacokinetic properties revealed that the compounds were likely non-carcinogenic, non-hepatotoxic, and rapidly soluble. Molecular dynamic (MD) modeling was used to identify the most favorable docked complexes involving bioactive chemicals. MD simulations indicate that varying types of kaempferol-O-rhamnoside are necessary for successful docking validation and maintaining the stability of the docked complex. These findings could facilitate the discovery of novel therapeutic agents for treating illnesses caused by the Mpox and MARV viruses.

## Introduction

In the middle of the severe acute respiratory syndrome coronavirus-2 (SARS-CoV-2) infection that emerged as a novel virus in 2019, and has since caused the ongoing coronavirus disease 2019 (COVID-19). Two additional zoonotic viruses, Monkeypox virus (Mpox) virus and Marburg virus (MARV), have recently re-emerged in the current years (Islam et al., 2023; Zaeck et al., 2023). With over 60,799 confirmed cases of Mpox reported from 99 countries and locations, along with 20 deaths as of September 16, 2022, the re-emergence of Mpox, a member of the family Poxviridae and genus Orthopoxvirus that also contains smallpox virus, causing monkeypox (Mpox) in humans, has posed a public health emergency of international concern (Mohapatra et al., 2022; Khattak et al., 2023). MARV, a member of Filoviridae family that also contains deadly Ebola virus, which causes Marburg virus disease (MVD), and has a fatality rate of up to 90%, re-emerged in Ghana, an African country, in July 2022, as well as it was also reported from Guinea in 2021; there have been a total of 15 MVD outbreaks, the vast majority of which have occurred in Africa (Abir et al., 2022; Aborode et al., 2022; Zhao et al., 2022). MARV was initially documented to infect people in 1967, while Mpox was first identified in 1958, with the first zoonotic human case confirmed in 1970. These two viruses have been rare and forgotten human diseases for almost 50 years. Among these, Mpox has recently become a global health emergency (Farahat and Memish, 2022; Anwar et al., 2023).

**Figure 1:**
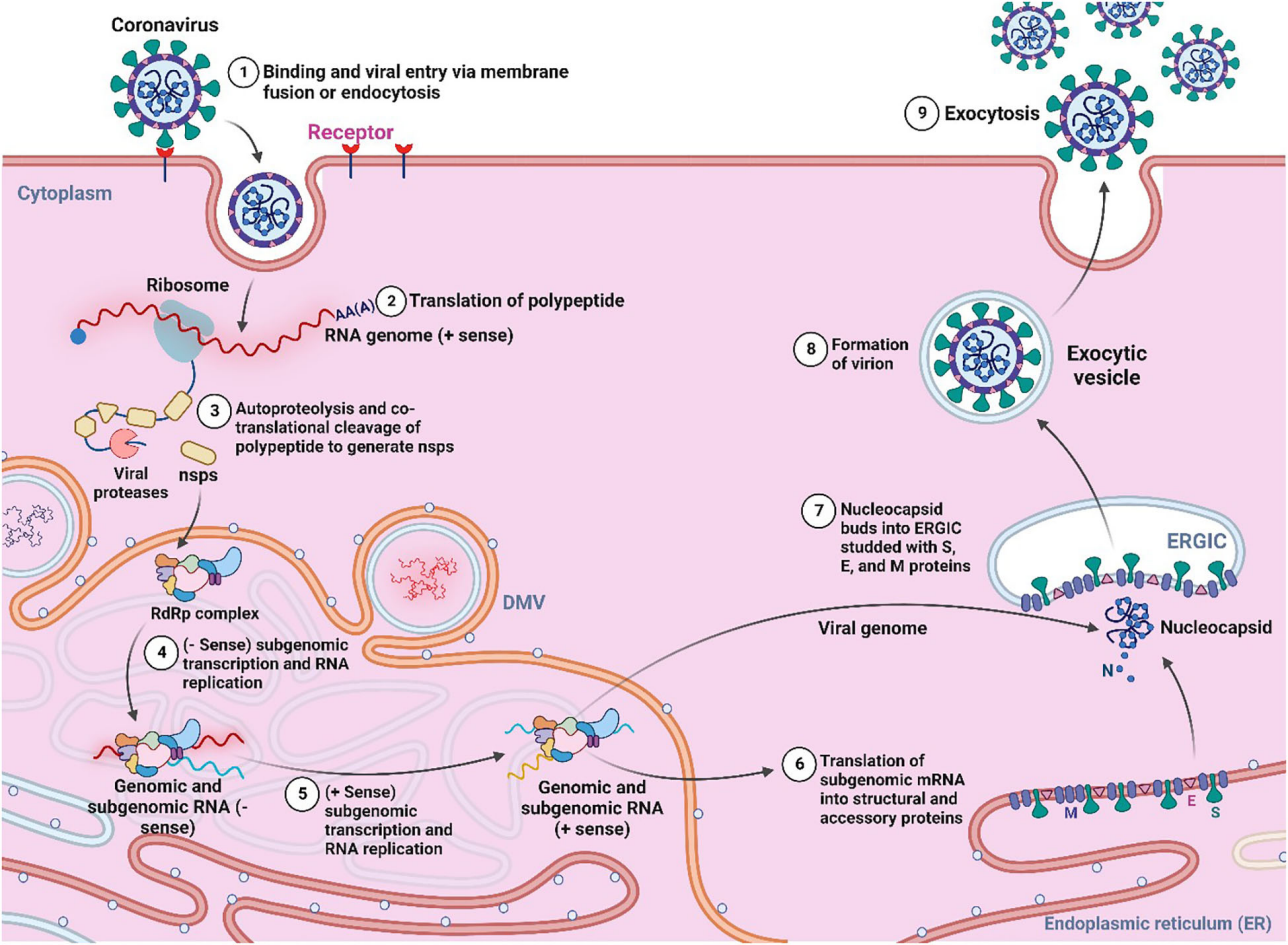
General pathogenesis and life cycle of virus (Created with BioRender.com).

The viruses are obligate intracellular parasites that can act as living, non-loving objects (Claverie, 2006). They are very tiny in size and have a simple structure. They have the lack any metabolic activity and consist of either DNA or RNA as a nucleic acid, protein, and membrane lipids which surround them. Generally, the proteins provide the structural shape of the virus, which include capsid proteins, matrix proteins, and membrane glycoproteins (Whittaker and Helenius, 1998; Claverie, 2006). Due to obligate parasites in nature, they can act as non-living objects outside of host cells, and when they reach the physiological system or any living host cell, they are able to cross cell membranes even several other barriers in order to transport their genetics into the cytosol or nucleus of the host cell (shown in **Figure 1**), which ultimately results in pathogenicity or infectious disease to the host cells (Marie, 1957). The viral infection spreads quickly, and host-cell propagation occurs in three primary stages: viral particle formation in infected host cells; virus escape into the extracellular space; and finally, virus entrance into a newly exposed cell membrane (Boulant et al., 2015). Diseases caused by viruses often start in the periphery of the body, and may migrate to the internal organs, liver, kidney, heart, gastrointestinal system, neurological system of mammals, where they can directly impact the vital organs, central nervous system (CNS) and the peripheral nervous system (PNS) (Becker, 2021).

According to the recent outbreaks and findings, viruses are still the cause of a vast variety of infectious illnesses in humans and animals, as well as a substantial proportion of newly developing and reemerging infections that are considered to be of significant health concern (Fauci, 2001). Some emerging/re-emerging notable pathogenic viral infections include SARS CoV-2 (Islam et al., 2022; Shanmugaraj et al., 2022), influenza viruses including bird flu virus (H5N1), Crimean-Congo hemorrhagic fever virus (CCHFV), Zika (ZIKAV), Nipah (NiV), Hendra virus (HeV), Ebola (Coltart et al., 2017), Monkeypox (Beer and Rao, 2019), Langya Henipavirus (Akash et al., 2022) and Marburg virus (Akash et al., 2023). Due to the rarity of Mpox, it has infected a huge number of people during the current COVID-19 pandemic still has a lot of unknowns (León-Figueroa et al., 2022; Taseen et al., 2022). Infections can spread from animals to humans, making this a zoonotic virus. Rapid human-to-human transmission has been primarily noticed with the recent re-emergence of Mpox in multiple nations, causing a worldwide health emergency. This virus is the most commonly found in Western and Central Africa (Chakraborty C. et al., 2022; Velavan and Meyer, 2022). The clinically significant illness produced by Mpox is comparable to those seen in smallpox but is not as devastating (Harapan et al., 2020; Khattak et al., 2022). This pathogenic virus was first reported in 1970 in an infant who had previously been affected by smallpox (Ladnyj et al., 1972). Despite the fact that this disease was discovered more than 60 years ago, it has lately been reported to infect SARS CoV-2 patients who have been hospitalized since the emergence of the COVID-19 pandemic (Shanmugaraj et al., 2022). Since January 2022, the World Health Organization has received reports of more than 47000 patients diagnosed with Mpox (Sharma et al., 2022). Researchers and healthcare providers are getting more concerned about the possibility of a newer pandemic of Mpox (Adalja and Inglesby, 2022; Ahmed et al., 2022). Secondly, MARV as another zoonotic pathogen has recently attracted attention of the scientists owing to its recent year’s outbreaks in Ghana and Guinea (Nyakarahuka et al., 2019). In Uganda, there were reported for three separate MVD epidemics in 2007, 2012, and 2014, and thereafter from other African countries and beyond Africa, 6-8 MVD epidemics were also identified (Adjemian et al., 2011; Knust et al., 2015; Nyakarahuka et al., 2017; Zhao et al., 2021). The MARV produces severe or even life-threatening effects owing to its very high case fatality rates (Balter, 2000; Zhao et al., 2021).

There are very few approved drugs, licensed vaccine, or therapeutic without vast ranging of potential activity yet available against both the Mpox and MVD, though efforts have been made, as well currently being advanced for finding out effective prophylaxis and treatment regimens for both of the diseases (Dulin et al., 2021; Chakraborty S. et al., 2022; Hickman et al., 2022; Sah et al., 2022). However, European medical agency has approved tecovirimat drug against Mpox (Ali et al., 2023). Many researchers are trying to develop potential medication from the natural sources against mentioned disease. Currently, a number of different drugs have been shown to be effective against the Mpox when tested *in vitro*, such as Adamantane derivatives, Resveratol, Tecovirimat, Mitoxantrone, and Ribavirin. Nevertheless, their usefulness and safety in the context of Mpox have not been shown, and further basic and clinical research is needed (Ali et al., 2023; Khan et al., 2023; Khani et al., 2023).

In addition to novel drugs, chemical ligands, broad-spectrum antivirals, and broadly neutralizing antibodies, it is worth noting that the prophylactic and therapeutic aspects of medicinal plants and herbs, their extracts and metabolites, phytochemicals, immunity-enhancing foods, and dietary nutrition have shown potentials for countering several important infectious pathogens and deadly viruses, including Mpox and MARV (Khan et al., 2023; Nguyen, 2023; Pourhajibagher and Bahador, 2023). O-rhamnosides, including Kaempferol-O-rhamnosides, are a class of polyphenolic compounds with a variety of beneficial biological effects, such as antimicrobial, antioxidant, and anti-inflammatory properties (León-Figueroa et al., 2022; Velavan and Meyer, 2022). Their propensity to bind with p-hydroxybenzoic acid in DNA may also explain why they suppress polymerase chain reaction. Virus replication is stifled as a result of this interaction, which blocks the transcription of viral DNA into RNA (Zhao et al., 2021). Therefore, Kaempferol-o-rhamnoside and its eight derivatives were chosen as the primary molecules in this investigation, and their antiviral capability was investigated against Mpox and MARV infections for their utility in treating patients with Mpox and MVD. Although these two pathogenic viruses have the feasible potential to cause another pandemic, it is unfortunate that no effective or potentially effective medication has been developed to date (Kortepeter et al., 2020). So, this investigation has been performed to identify a potential medication which may inhibit Mpox and Mpox with lower adverse effects by using Computer-Aided Drug Design (CADD) techniques and quantum chemistry of chemical descriptors. In comparison to traditional procedures, these strategies have been shown to be productive across the whole drug development process, resulting in a reduction in the amount of money and time required to produce a medicine (Rahman et al., 2012).

## Materials and methods

### Optimization and ligand preparation

O-rhamnoside is a type of carbohydrate and contains the active methyl group where one, two and three groups are attached to form the L02, L03, and L04 compounds. Kaempferol, on the other hand, is a phytochemical derived from the *Talipariti elatum* tree and was employed as the mother compound in this investigation. Next, the Kaempferol is attached with L01, L02, L03, and L04 to form L06, L07, L08 and L09 as derivatives of Kaempferol-o-rhamnoside.

DFT functional methods were employed towards complete molecular optimization using DMol3 code from Material Studio 08 package (Kumer and Khan, 2021). To ensure accuracy, the DMol3 code was put up using the functional of B3LYP and Gaussian double zeta plus polarization function basis set (DNP). Following geometric optimization, the HOMO and LUMO molecular border orbital diagrams were split, and calculated the electron affinity, electron negativity, energy gap, chemical potential, hardness, softness, and electrophilicity index using the following equations (a-h), respectively. Finally, the optimized molecule was stored as a PDB file for future in-silico investigation, such as molecular docking, molecular dynamics, and ADMET analysis.


$$\begin{array}{c} {\text{E}}_{\text{gap}}\;=({\text{ E}}_{\text{LUMO}}-{\text{E}}_{\text{HOMO}})\ldots \ldots \ldots \ldots \ldots .\left(\text{a}\right);\;I=-E_{HOMO}\ldots \ldots \ldots \ldots .\ldots .(b)\\ A=-E_{LUMO}\ldots \ldots \ldots \ldots (c);\;(x)=\frac{I+A}{2}\mathrm{\ldots \ldots \ldots \ldots .}(d);(\omega )=\frac{\mu^{2}}{2\eta }\mathrm{\ldots \ldots \ldots \ldots .}(e)\\ (\mu )=\frac{I+A}{2}\ldots \ldots \ldots \ldots (f);(\eta )=\frac{I+A}{2}\mathrm{\ldots \ldots \ldots .}(g);\\ \left(s)=\frac{1}{\eta }\ldots \ldots \ldots \ldots (h\right) \end{array}$$

### termination of the data of ADMET and lipinski rule

It is critical to forecast ADMET traits during the discovery or development of a new pharmaceutical in order to prevent treatment failure throughout clinical investigations (Cheng et al., 2013). As a consequence, in-silico pharmacokinetic prediction plays a significant role. The pkCSM was utilized to create this prediction so that there was less risk of the new drug molecules failing during the trial phases and greater possibility of them making it to the final step as prospective drug candidates (Adalja and Inglesby, 2022). This prediction is based on key factors, such as molecule uptake in the human intestine, percolation capabilities of the blood-brain barrier, central nervous system, metabolism capabilities, total clearance, bioavailability, and toxicity levels.

### PASS prediction

The PASS prediction has been made to determine the capability of the ant-viral drug. The online web program PASS (http://www.pharmaexpert.ru/passonline/) was used to predict the anti-bacterial, anti-fungal, and anti-parasitic spectrum (Lagunin et al., 2000). The configurations of Kaempferol-o-rhamnoside derivatives were illustrated first, and then they were transformed into smile forms by the addition of the free online programs provided by SwissADME (http://www.swissadme.ch) (Daina et al., 2017). These programs are well-known for their ability to determine PASS spectrum by making use of the PASS web tool. PASS findings are represented by the probabilities Pa (probability for an active molecule) and Pi (probability for an inactive molecule). Pa and Pi grades may vary from 0.00 to 1.00, and Pa and Pi must be less than 1, since potentialities can be anticipated in whatever way the researcher chooses. The drug candidate should be potential and bioactive if the score of Pa > Pi (Poroikov and Filimonov, 2005).

### Preparation of protein and method for molecular docking

**Figure 2:**
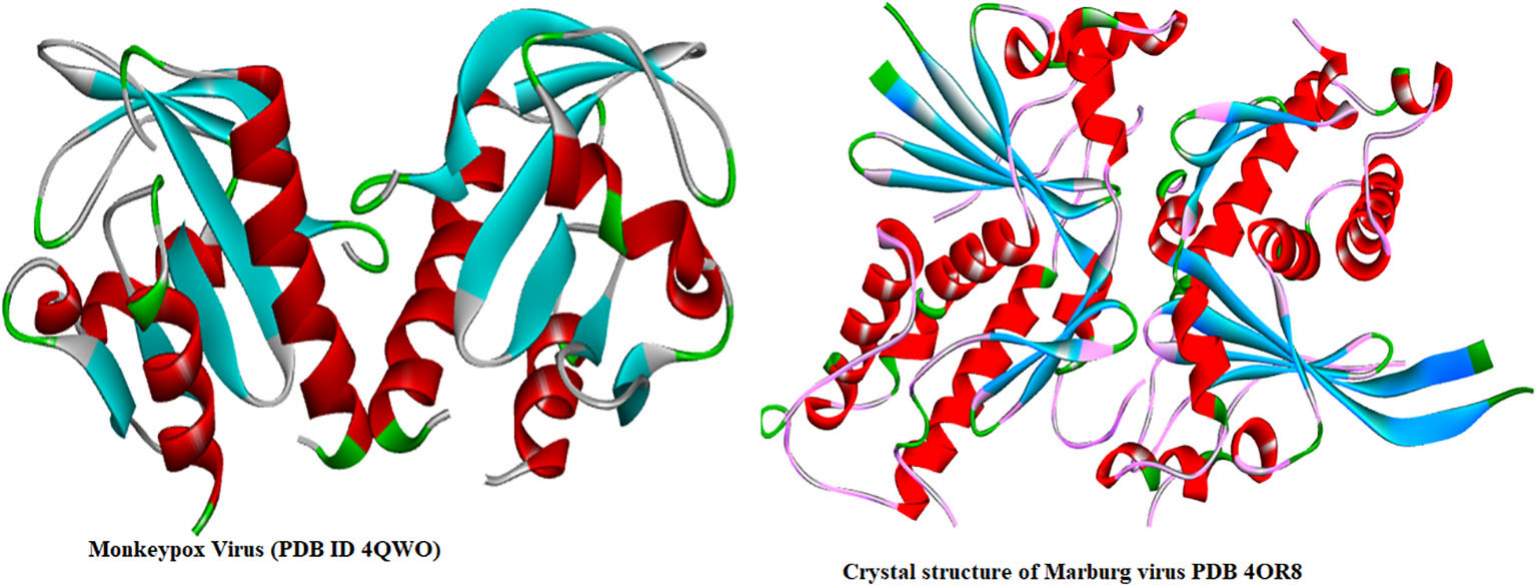
Three-dimensional protein structure of Monkeypox and Marburg viruses (Zhang et al., 2014; Minasov et al., 2022).

Molecular docking is one of the most effective ways of virtual screening, particularly when there is a computer-based drug design and three-dimensional structure of the receptor and ligand accessible. Firstly, two targeted receptor proteins, such as Mpox (PDB ID: 4QWO), and MARV (PDB ID: 4OR8) were download from Protein Data Bank (PDB) (https://www.rcsb.org/) which were isolated by X-ray diffraction method with high stable configuration. After that, PyMol version 2021 was applied for removing all the excess heteroatom and minimized the energy by Swiss PDB viewer (Joseph and Namboothiri, 2015; Mooers and Brown, 2021). Finally, docking analysis was completed with the help of AutoDock tool and during this progres (Dallakyan and Olson, 2015), the grid box parameter was used as center X =12.4697, Y =15.9818, Z=16.0634, and Dimension (Å) X = 35.144, Y= 37.645Å, and Z= 36.966 Å for Mpox (PDB ID 4QWO). In case of MARV, center is in X = 14.267 Å, Y = 4.929, Z= 14.557, and Dimension (Å) X = 53.382 Å, Y= 48.667 Å, and Z= 66.040 Å. Three-dimensional structures of Mpox and MARV proteins are displayed in **Figure 2**.

### Molecular dynamics simulation

On a high-configuration computer, molecular dynamics simulation was used in live view according to the capabilities afforded by NAMD (Kumer et al., 2022b). The docking results for the most powerful drugs were validated by molecular dynamics simulations up to 100 nanoseconds for the hollow tube (drug-protein). In addition, NAMD is particularly well suited to the increasingly popular Beowulf-class PC clusters, which are quite similar to the workstation clusters for which it was originally designed. MD simulation determines the stability of molecules and verifies the docking fitting. In this study, the MD simulation was performed at 100ns and applying AMBER14 force field (Nath et al., 2021). Before that, the whole system was in the presence of a water solvent equilibrated with 0.9% NaCl at 298 K temperature in presence of liquid system with NVT ensemble having the simulation box size as boundary 1.5 and X=5, Y=5 and Z=5 which is standard for these proteins for solvation, as well as, its vector coordinate of box is at -25.06, 18.79 and -7.06, respectively. VMD evaluated the performance of this result using RMSD and RMSF. Next, the Ramachandran Plot and B-factor were evaluated to explain the validation and stability of docked complex.

## Results and analysis

### Chemistry of O-rhamnosides and Kaempferol-O-rhamnosides derivatives

**Figure 3:**
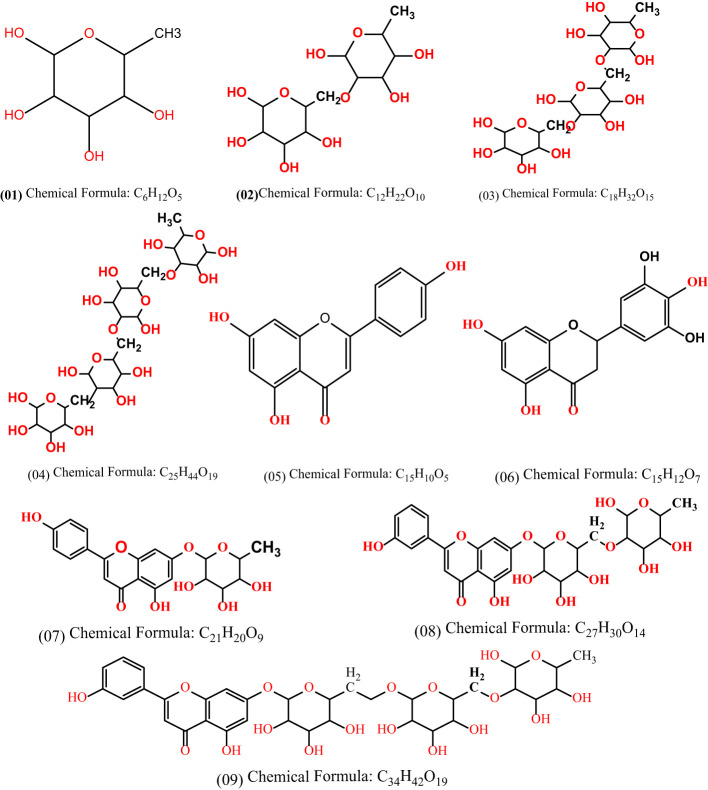
Chemical structure of derivatives of O-rhamnosides and Kaempferol-O-rhamnosides.

In case of O-rhamnoside (L01), it is revealed that the L02, L03 and L04 are formed by attaching one, two and three O-rhamnoside rings with the original compound of O-rhamnoside, as shown in **Figure 3**. Moreover, the compound L01, L02, and L03 is attached with Kaempferol to form L07, L08 and L09 compounds. Finally, the computational and in silico analysis as well as SAR and comparative studies are performed on them.

### Optimized structure of derivative of O-rhamnosides and Kaempferol-o-rhamnosides

**Figure 4:**
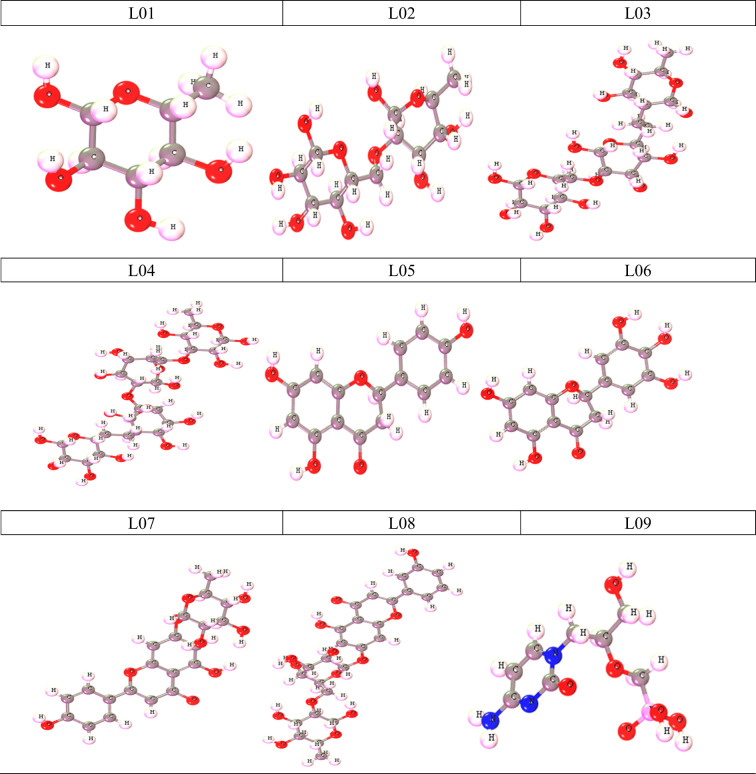
Optimized structure of derivative of O-rhamnosides and Kaempferol-o-rhamnosides.

In computational chemistry, the use of quantum mechanical methods for the calculation of thermodynamic, molecular orbital, and molecule electrostatic characteristics is prevalent. The program known as Gaussian 09 has been run in order to improve the geometry and further enhance each generated derivative. The density functional theory (DFT), functional of B3LYP and Gaussian double zeta plus polarization function basis set (DNP), was utilized in order to improve and predict the molecular orbital and thermal properties of the molecules. All compounds had their dipole moment, enthalpy, free energy, and electronic energy determined. The optimized molecular structures are shown in **Figure 4**.

### PASS prediction

The probable biological spectrum for Kaempferol-o-rhamnoside derivatives has been predicted by applying the web server PASS. The PASS data is summarized as Pa and Pi, which are shown in **Table 1**. According to the presupposition in **Table 1**, Kaempferol-o-rhamnoside derivatives 02–09 demonstrated 0.379<Pa< 0.712 for antiviral (Influenza), 0.423<Pa<0.655 for antibacterial, 0.632< Pa< 0.755 for antifungal, and 0.214<Pa<0.391 for anti-parasitic, while Ligand 07 demonstrated Pa 0.634<pa. Consequently, the PASS assessment showed that 0.379< Pa< 0.712 for antiviral (Influenza), which indicated that the Kaempferol-O-rhamnoside derivatives have a greater potential as antiviral agent compared to other antibacterial and anti-parasitic characteristics. Although the antifungal Pa score is higher, it has been largely ignored in favor of the antiviral Pa score since researchers are more interested in discovering new antiviral drugs.

**Table 1:** Data of PASS prediction.

Data of PASS Prediction								
Ligand No	Anti-viral (Influenza)		Anti-bacterial		Anti-fungal		Anti-parasitic	
	Pa	Pi	Pa	Pi	Pa	Pi	Pa	Pi
L01	0.659	0.009	0.571	0.011	0.632	0.015	0.214	0.095
L02	0.633	0.010	0.589	0.009	0.657	0.013	0.371	0.037
L03	0.463	0.029	0.623	0.028	0.735	0.008	0.377	0.064
L04	0.415	0.042	0.643	0.077	0.750	0.007	0.290	0.059
L05	0.633	0.010	0.589	0.009	0.657	0.013	0.371	0.037
L06	0.712	0.005	0.423	0.025	0.604	0.018	0.365	0.039
L07	0.700	0.005	0.629	0.007	0.752	0.007	0.634	0.008
L08	0.579	0.015	0.635	0.007	0.755	0.007	0.391	0.033
L09	0.379	0.053	0.655	0.006	0.751	0.007	0.216	0.090

### Lipinski’s rule, pharmacokinetics and drug likeness

Due to a variety of factors, the most medication candidates never become commercially available or cannot pass the clinical or preclinical stages. It is the utmost significance to create trustworthy computational approaches for the estimation of the drug-likeness of new drug candidates that increase the percentage of successful drug discovery and development attempts during the trial phase. The drug-likeness prediction model was developed by Christopher A. Lipinski in 1997, which included molecular descriptors connected to the numbers of factors that can differentiate between possible drugs and non-drugs. These factors include topological polar surface area, number of rotatable bonds, hydrogen bond acceptor, hydrogen bond donor, and molecular weight. The molecular weight of the reported Kaempferol-o-rhamnoside derivatives (L01-L09) was at 164.16-754.69, the number of rotatable bonds was 0.0-10, the hydrogen bond acceptor was 05-19, the hydrogen bond donor was 04-13, and the topological polar surface area was 90.15Å² -318.37Å². So, only Ligand of L01, L02, L06, and L07 satisfied the overall Lipinski rule (as shown in **Table 2**). However, the other molecules do not satisfy because they have a larger topological polar surface area, have a hydrogen bond acceptor, or are a hydrogen bond donor. Finally, the bioavailability score for ligands L01, L02, L04, and L05 is greater than 0.55, and the remaining molecules’ bioavailability scores are reported at 0.17. So, the topological polar surface area or hydrogen bond acceptor and hydrogen bond donor have been abandoned.

**Table 2:** Data of lipinski rule, pharmacokinetics and drug likeness.

Data of Lipinski rule, Pharmacokinetics and Drug likeness								
Ligand No.	Molecular weight	Number of rotatable bonds	Hydrogen bond acceptor	Hydrogen bond donor	Topological polar surface area (Å²)	Lipinski’s rule		Bioavailability Score
						*Result*	*Violation*	
L01	164.16	00	05	04	90.15	Yes	00	0.55
L02	326.30	03	10	07	169.30	Yes	01	0.55
L03	486.46	06	14	10	239.29	No	02	0.17
L04	648.61	09	19	13	318.37	No	03	0.17
L05	488.44	06	15	10	248.45	No	02	0.17
L06	304.25	01	07	05	127.45	Yes	00	0.55
L07	416.28	03	09	05	149.82	Yes	00	0.55
L08	578.82	06	14	08	228.97	No	03	0.17
L09	754.69	10	19	11	308.12	No	12	0.17

### Quantum calculation and HOMO-LUMO and frontier molecular orbital (FMO)

**Figure 5:**
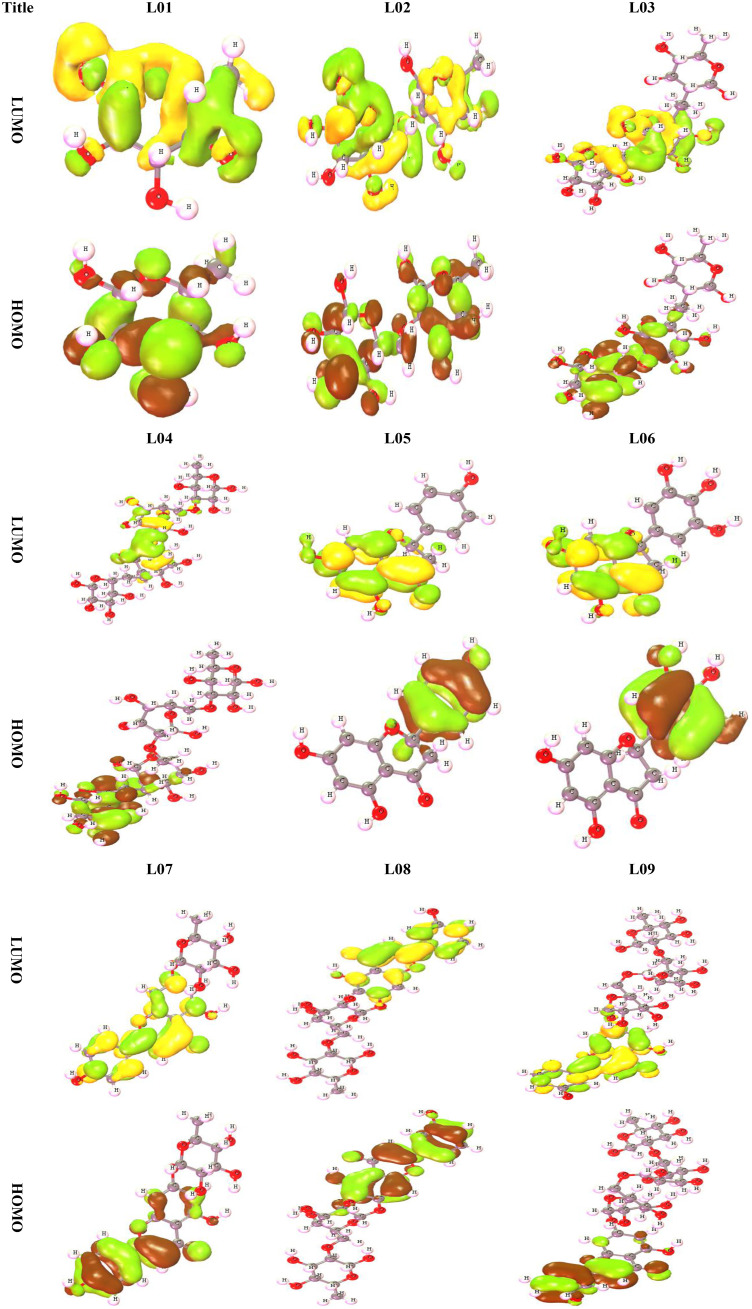
Frontier Molecular orbital for derivative of O-rhamnosides and Kaempferol-o-rhamnosides.

Frontier molecular orbitals (FMO) are the most important orbitals in a molecule. They are used by scientists to study chemical reactivity and kinetic stability. People often talk about the highest occupied molecular orbital (HOMO) and the lowest unoccupied molecular orbital (LUMO) when talking about these molecular orbitals on the edge of the atom (LUMO). When you say “electronic absorption,” you mean a change from the ground state to the first excited state. This is usually thought of as one electron moving from the HOMO state to the LUMO state (Perepichka and Bryce, 2005; Kumer et al., 2021a). A molecule’s high level of chemical stability and low level of chemical reactivity are directly linked to how long its energy difference lasts (Kumer et al., 2022a). Because it makes it easier for electrons to move from one electron to another, a low energy gap is linked to low chemical stability and high chemical reactivity. Because of this, it takes a lot more energy for an electron to move from its ground state, called HOMO, to its excited state, called LUMO. **Table 3** highlights the HOMO and LUMO energies, the HOMO-LUMO gap (Δ), and the hardness (η) and softness (S) indexes of all derivatives of Kaempferol-o-rhamnoside, while **Figure 5** highlights, for better understanding, the frontier orbital diagrams of both HOMO and LUMO, as shown using a variety of color schemes to facilitate better comprehension. In the context of HOMO, the shade of deep radish signifies the positive nodes of orbitals, while the shade of yellow corresponds to the negative node of orbitals.

**Table 3:** Data of chemical descriptors.

Data of chemical descriptors								
S/N	A=-LUMO	I=- HOMO	Energy =I-A	Chemicalpotential	Electronegativity	Hardness	Softness	Electrophilicity
L01	-1.377	-10.901	9.524	6.139	-6.139	4.762	0.2100	-3.957
L02	-1.461	-10.772	9.311	6.116	-6.116	4.655	0.2148	-4.018
L03	-1.184	-10.279	9.095	5.731	-5.731	4.547	0.2199	-3.611
L04	-0.954	-10.257	9.303	5.605	-5.605	4.655	0.2150	-3.377
L05	-1.219	-8.868	7.649	5.043	-5.043	3.824	0.2615	-3.325
L06	-1.276	-9.033	7.757	5.115	-5.115	3.878	0.2578	-3.425
L07	-1.590	-8.607	7.017	5.098	-5.098	3.508	0.2850	-3.704
L08	-1.828	-8.783	6.995	5.305	-5.305	3.477	0.2876	-4.047
L09	-1.660	-8.617	6.957	5.138	-5.138	3.478	0.2875	-3.795

As indicated in **Table 3** and **Figure 5**, the energy gap value of Kaempferol-O-rhamnoside derivative 01-04 is consistently larger (9.524, 9.311, 9.095 and 9.303 eV) compared to other considered molecules. The range of chemical hardness lies between 4.762 and 3.477, while the softness values of the Kaempferol-O-rhamnoside derivatives extend from 0.2100 to 0.2876. It's noticeable that hardness and softness share a reciprocal relationship; a decrease in hardness consequently corresponds to an increase in softness. In the case of ligands, the least hardness is observed alongside the highest softness.

From the **Figure 5**, it is observed that the LUMO is scattered throughout in whole molecules for L01 and L02, but it is different for the other ligands (L03-L09). The LUMO is stayed in the O-rhamnoside for L01 to L09 but the HOMO is found in the kaempferol ring.

### Molecular electrostatic potential charge distribution mapping

**Figure 6:**
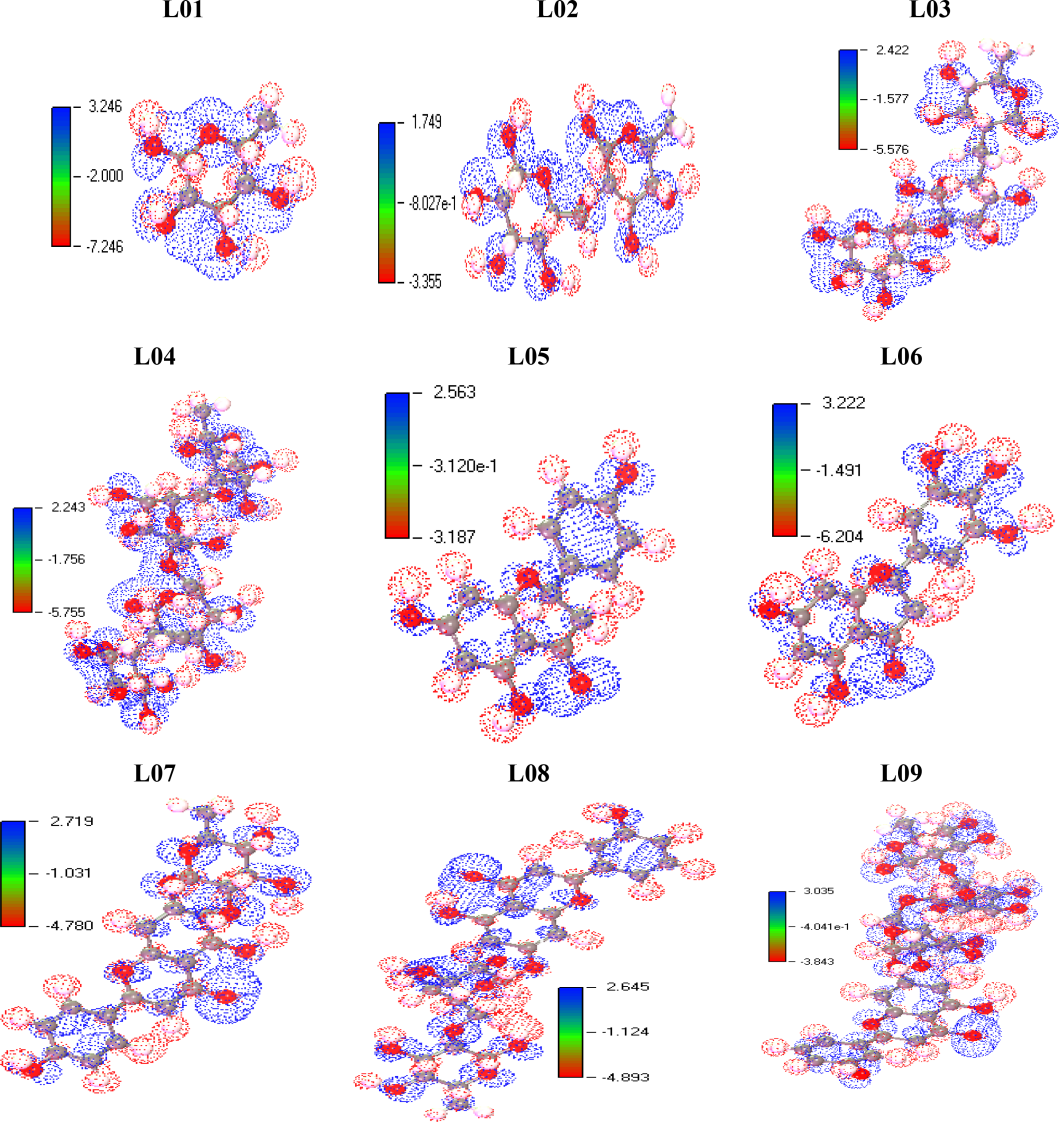
MEP mapping for the ligands after DFT optimization.

The Molecular Electrostatic Potential, also known as MEP, is utilized as a global reactivity map. This map illustrates the amount of an organic molecule that is most equipped for the electrophilic and nucleophilic onslaught of charged point-like reagents (Rahman et al., 2022). It is helpful in understanding the biological recognition system and the coupling of hydrogen bonds. This MEP counter map offers a straightforward method for forecasting how various types of geometry could interact with one another. The significance of molecular electrostatic potential (MEP) arises from the fact that it concurrently demonstrates a molecular shape and size and also positive, negative, and neutral electrostatic potential provinces in terms of visual assessment. In addition to this, MEP is very effective in the investigation of macromolecules with physical and chemical features. On the basis of the optimized structure of the Kaempferol-O-rhamnoside derivatives (L01-L09), MEP was utilized to make a prediction about the active sites on the surface that would be susceptible to electrophilic and nucleophilic interactions. The different electrostatic potential has been shown by the red and blue hue. The red color indicates the maximum negative area, which is a significant site for electrophilic interactions; the blue color represents the largest positive region, which is an acceptable site for nucleophilic interactions; and the green color represents the neutral potential area. (See **Figure 6**).

### Molecular docking

The forces between molecules that occur between the ligand and the receptor during the formation of a protein-ligand complex are called binding affinity. This method has grown in popularity as a result of the research and investigation of innovative drug design (Meng et al., 2011). A binding energy of -6.00 kcal/mole has been speculated as a potential drug candidate (Kumer et al., 2021b). In this experiment, the Auto-Dock Vina program was used to investigate a set of Kaempferol-o-rhamnoside derivatives by the in-silico method in order to emphasize their putative binding energy and interaction configurations with the targeted protein of Mpox (PDB: 4QWO) and MARV (PDB: 4OR8). In light of the findings that were acquired from the docking inspection, the three derivatives with the highest binding energies were found. The results of computed analysis showed that three derivatives L07, L08, and L09 had potential docking scores of -9.2 (> -6.0), -9.0(>-6.0) and -9.4(>-0.6) against protein Mpox (PDB: 4QWO). Also, these three derivatives L07, L08, and L09 had potential docking scores -8.1 (> -6.0), -8.4(>-0.6) and -9.0(>-0.6) against Crystal Structure of the MARV (PDB: 4OR8). Where the traditional drug Cidofovir had the docking score -6.0(=-6.0) and -5.2(<-6.0) against protein Mpox (PDB: 4QWO) and Crystal Structure of the MARV (PDB: 4OR8) respectively. It demonstrates that L07, L08, and L09 substances can firmly bind to the protein receptors’ pockets than Cidofovir (as shown in **Table 4**). Besides, it is seen that by increasing the side chain, the binding energy is equally increased. It has been said that compared with standard antiviral drugs like Cidofovir, Ligand 03–09 shows better effectiveness.

**Table 4:** Binding affinity (kcal/mol) against Monkeypox and Marburg virus.

Drug Molecules No.	Binding Affinity(kcal/mol) against *Monkeypox and Marburg virus*	
	*Monkeypox virus (4QWO)*	*Crystal structure of Marburg virus (4OR8)*
L01	-5.6	-5.0
L02	-6.2	-5.8
L03	-8.0	-6.4
L04	-8.1	-7.0
L05	-7.4	-6.4
L06	-7.5	-6.7
L07	-9.2	-8.1
L08	-9.0	-8.4
L09	-9.4	-9.0
Cidofovir(D1)	-6.0	-5.2

### Protein-ligand interaction

**Figure 7:**
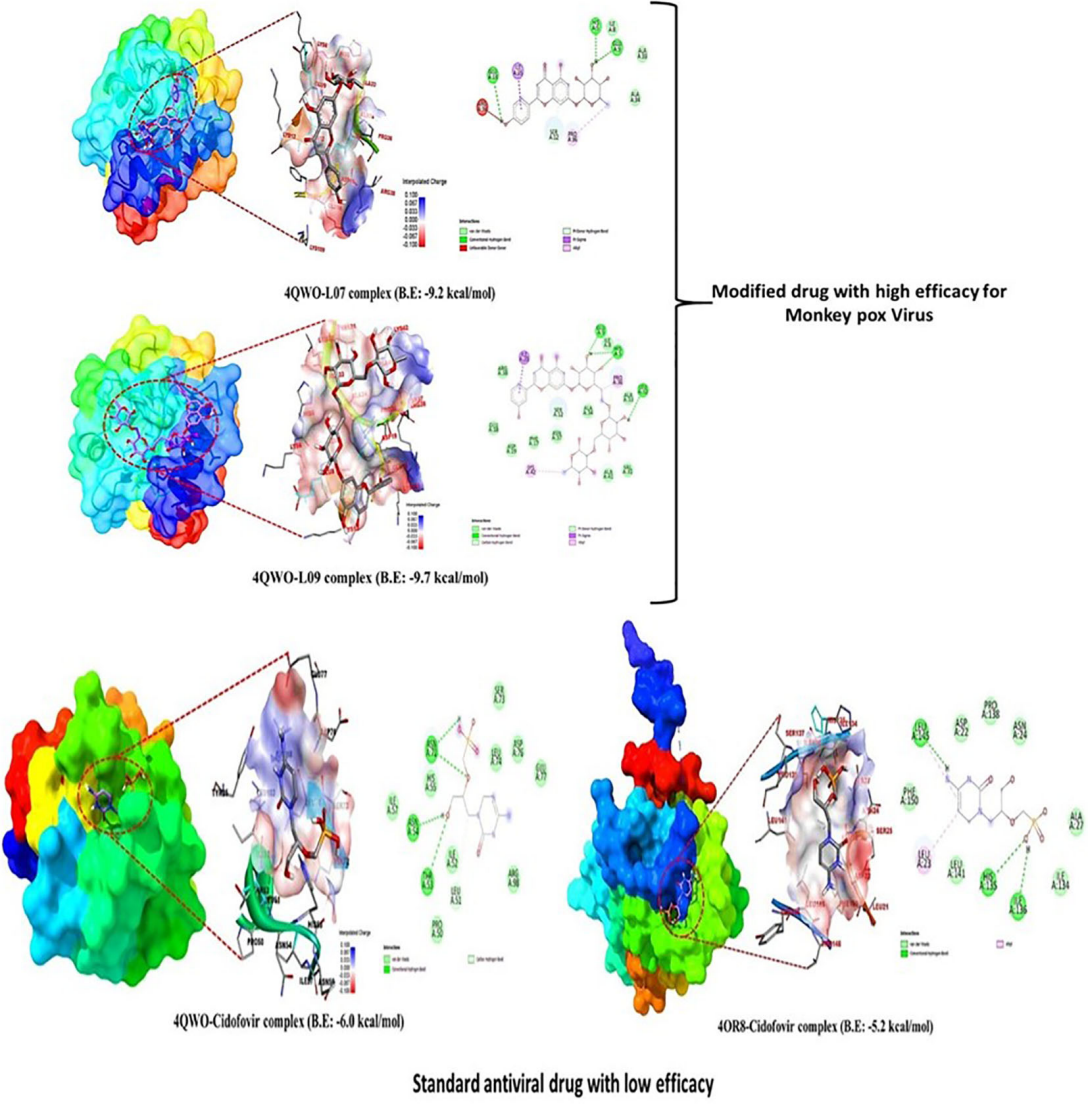
Lead picture of ligand and standard.

The Kaempferol-o-rhamnoside derivatives generated a wide variety of different types of non-covalent interactions with the active sites of the microbial proteins. These observed bonds included pi-alkyl, pi-anion, pi-sigma, amide pi-stacked, and pi-donor hydrogen bonds. Based on these results, it is abundantly obvious that aromatic substituent may quickly enhance the binding ability of uridine esters as well as their potential to inhibit the growth of reported viral pathogens due to the high electron density of aromatic substituent. The influence of H-bonds had a significant impact on the selectivity of ligand binding to the receptor, drug design in chemical and biological processes, and molecular recognition and biological activity. Therefore, the H-bond surface of all derivative ligands that interact with both proteins is presented in **Figure 7**. The 2D structure of protein ligand interaction also illustrates how to determine how many bioactive sides are present.

### Molecular dynamics simulation

**Figure 8:**
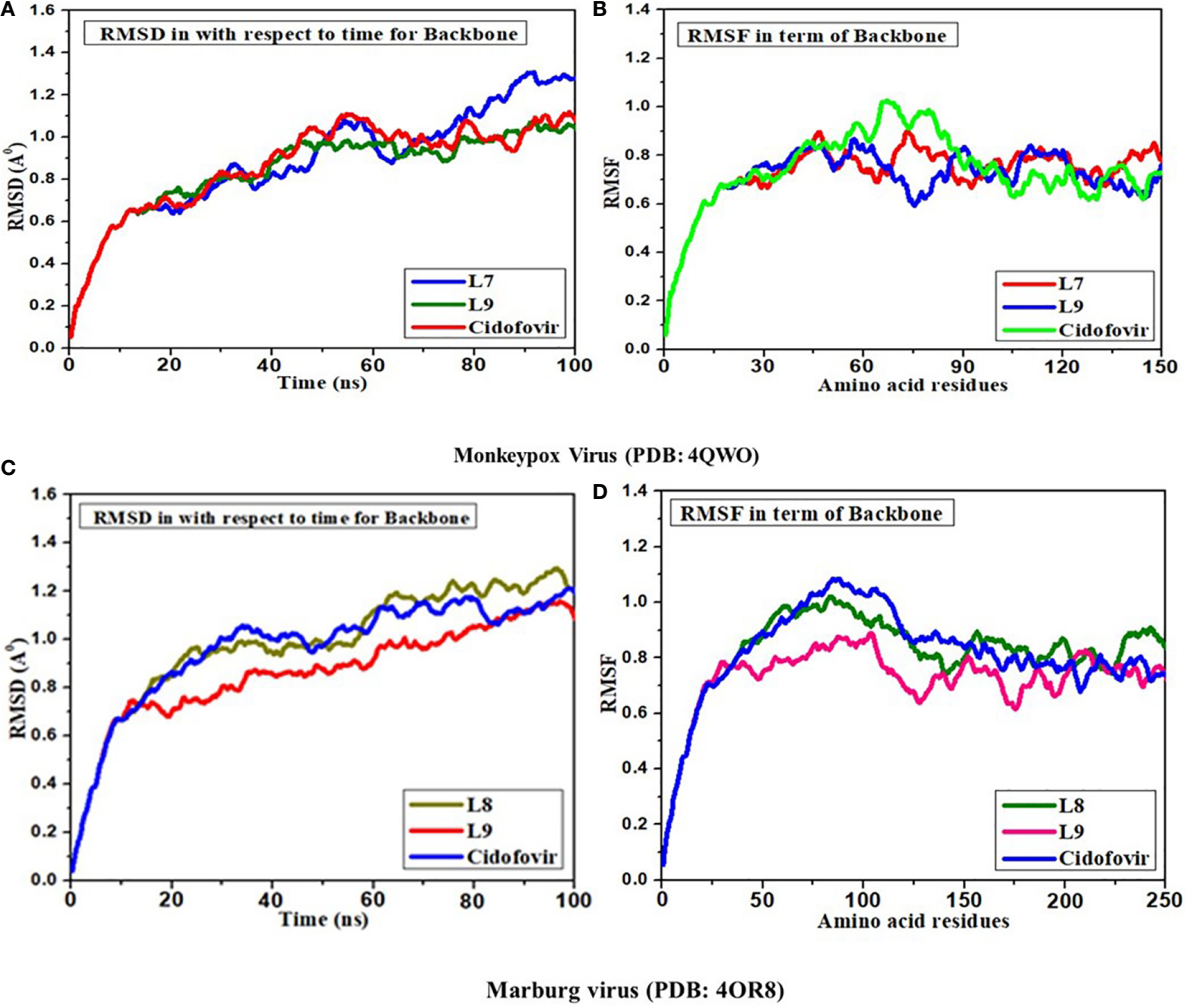
Analysis of **(A)** RMSD; **(B)** RMSF for L07, L09 and Cidofovir drugs in complex with Monkeypox virus **(C)** RMSD and **(D)** RMSF for L08, L09 and Cidofovir drugs in complex with Marburg virus.

Molecular dynamics (MD) simulations are effective tools for comprehending the physiological underpinnings that encompass the structure and function of bioactive molecules (Barroso da Silva et al., 2020). The initial conception of proteins as relatively inflexible arrangements has been superseded by a dynamic model according to which the internal movements of macromolecules and the conformational changes that follow from those movements play a significant key role in the development of new drug molecules. This MD simulation is a helpful tool for predicting the stability of drug-like molecules (Hickman et al., 2022). The outcome of MD simulation is described by RMSD and RMSF, which mean root-mean-square deviation and root-mean-square fluctuation. Stable molecules are biological molecules with a RMSD and RMSF score of less than 2.0 A° (Kawsar et al., 2022). So, based on maximum docking outcomes, Ligands of L07, and L09 have been conducted in MD simulation as well as standard Cidofovir (D1). According to the finding of MD simulation against Mpox (PDB ID 4QWO), the RMSD is about 0.9 Å –1.2 Å and similar outcomes were found in terms of RMSD in terms of backbone with amino acid residues. But, in RMSF, little impact has been seen in the addition of backbone with amino acid residues and there is fluctuation 0.7 Å – 0.8 Å (as shown in **Figures 8A, B**).

Secondly, the MD simulation has been completed for ligands 08 and 09 towards the MARV (PDB 4OR8). In this case, the RMSD and RMSF have been obtained with equivalent outcomes, and the RMSD is about 0.9 Å – 1.2 Å both time vs. backbone and backbone with amino acid residues, while the RMSF score at 0.7 Å – 0.8 Å (as shown in **Figures 8C, D**). In summary, the RMSD and RMSF scores are accepted and less than 2.00 Å which means these drugs could be stable after reaching biological systems.

### Ramachandran plot from molecular dynamic study

**Figure 9:**
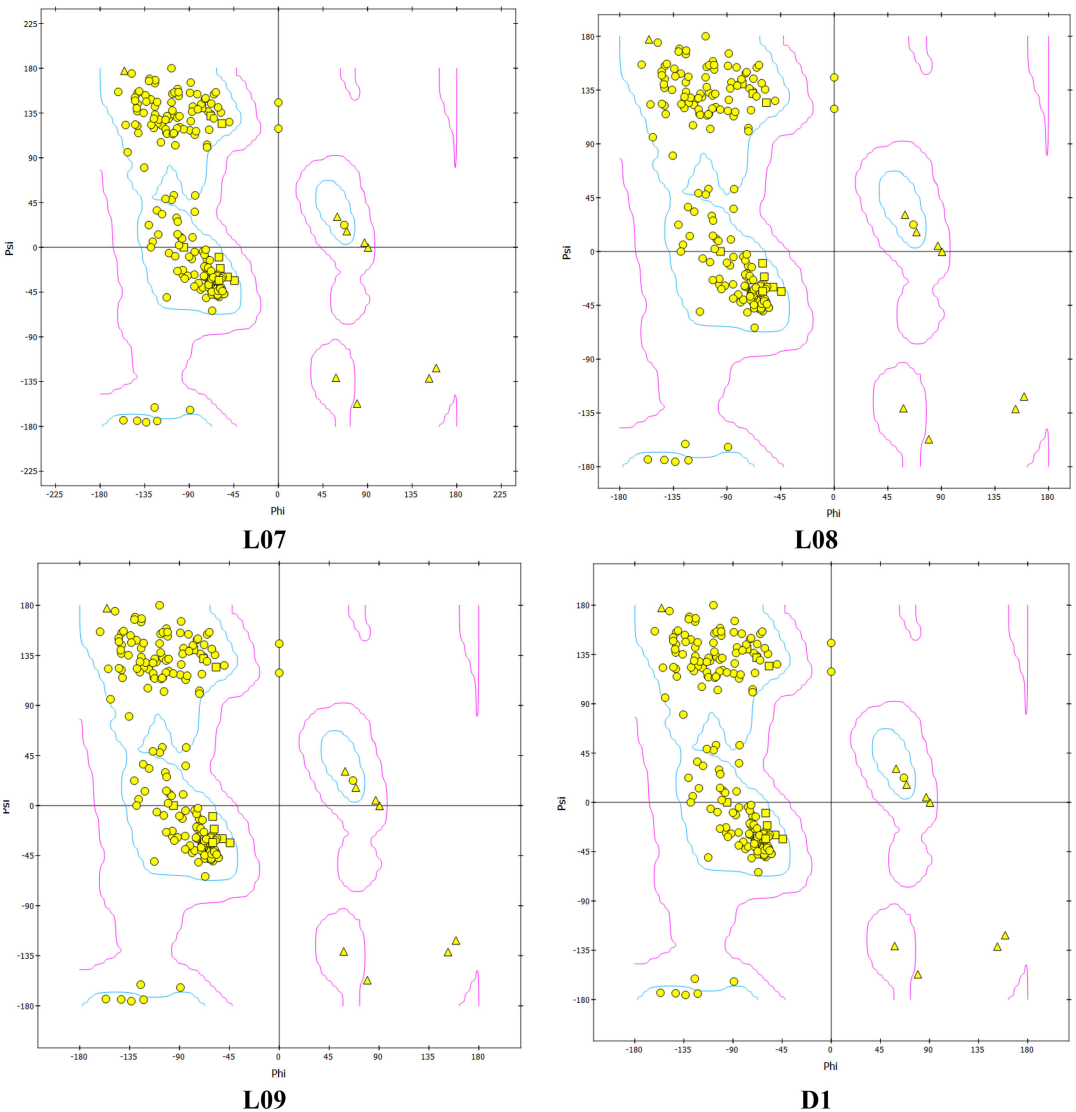
Ramachandran Plot from Molecular Dynamic Study for L07, L08, L09 and D1 for Monkeypox virus.

The Ramachandran plot is one of the most important parameters for checking chemical stability of the protein or enzyme structure satisfying both stereochemistry and steriochemical structure. In addition, it is also used for validation of docking and stability of docked complex of protein ligand. Also, this analysis illustrates the energy minimization of Botherombin and most favorable region. From the **Figure 9**, the Ramachandran plot is showed for the top three drugs (L07, L08 and L09), and compare with standard drugs (D1). There are 225 amino acids rasidues where 216 amino acid residues (96.0%) are out of favorable region, and 9 amino acids (4.00%) in unfavorable region for L07 which is almost similar trend for the L08, L09 and D1, means that there are no change after entering the ligand in protein. So that, it can be concluded that the molecular docking is valid and highly stable docked complexes were obtained after docking.

### B-factor in case of normal mode analysis (NMA) from molecular dynamic study

**Figure 10:**
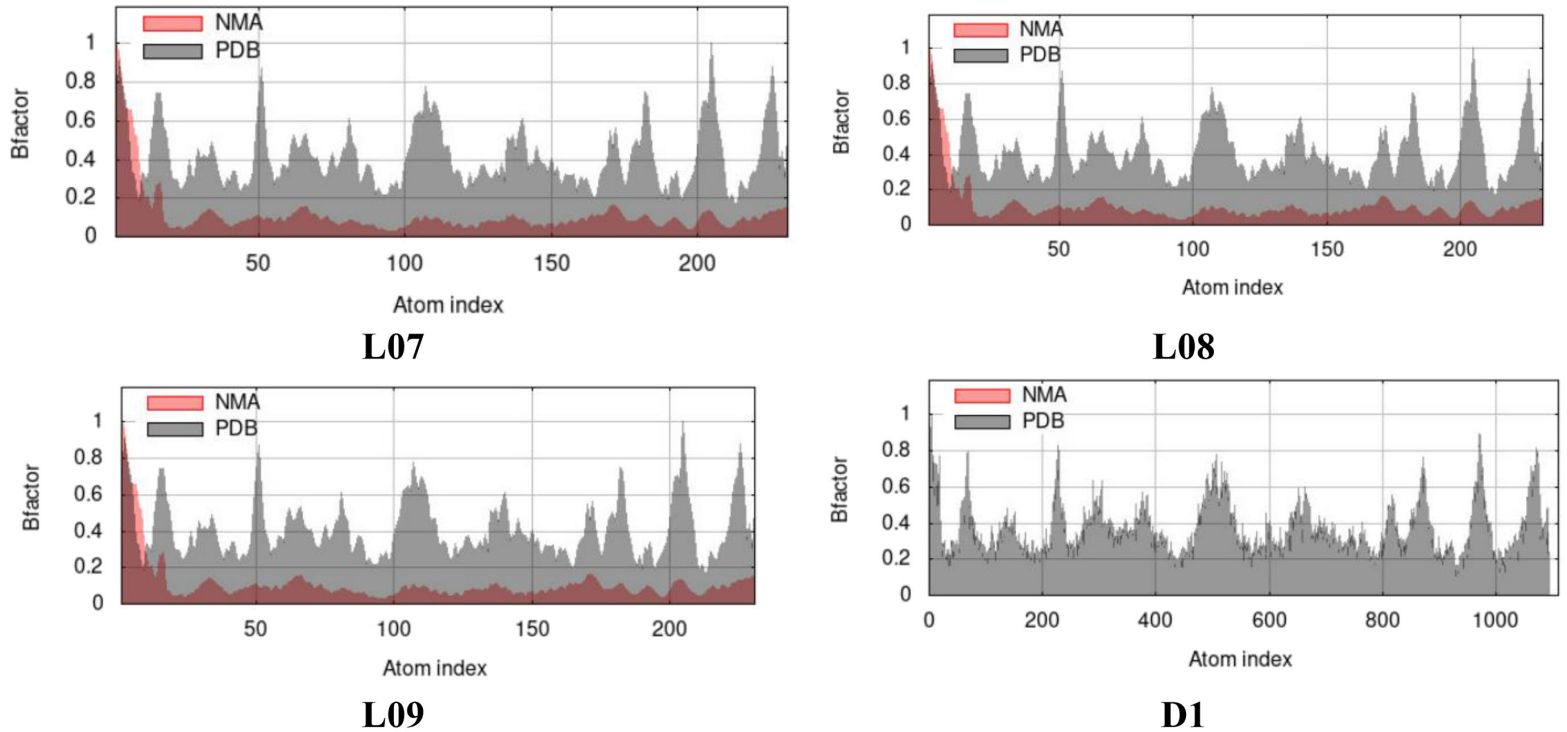
B-factor from Molecular Dynamic Study.

The expression B-factor, occasionally entitled the Debye-Waller factor, temperature factor, or atomic displacement parameter is to show by curve. In gerenal, the most significance of it is to express the higher flexibility results in larger displacements or lower electron density, indicating the lower stability. So after molecular docking, the B-factor of docked protein complex can be calculated that helps to say its deformation or stability at each of its residues by Normal Mode Analysis (NMA) of protein. From the **Figure 10**, the NMA of docked complex has been shown where the value of NMA is almost 0.8 or less than 1.0 for all residues for L07, L08, L09, and D1, that ensure the stability and validation of docking procedure.

### ADME and toxicity data

The present system of advanced manufacturing drug development includes the use of in silico techniques as an essential element. These tools are used to explore the absorption, distribution, metabolism, and excretion (ADME-PK) features of novel chemical substances. So, this ADMET analysis was performed to examine both the pharmacokinetic (PK) features and the toxicity of the compound. **Table 5** provides an overview of the compiled data for ADMET prediction. The Water solubility Log S standard score is considered to be from -4.0 to -6.0 and -2.0 to -4.0, respectively, for minimum and maximum solubility substances (Rout et al., 2022). Our finding log S is predicted to be -0.152 to -3.543, which falls in the range of maximum aqueous solubility. Substances (L01, L04, L06, L07, and L08) have a high rate of absorption, according to the results of the human intestinal absorption test (HIA) (HIA of more than 30% is considered a high absorption rate) (Pires et al., 2015). Additionally, the Caco-2 cell permeability measure was used to assess the absorption of the substances that were investigated. According to the findings, only three of the molecules exhibit Caco-2 cell permeability in a favorable manner. The VDSs (human) have been obtained from -0.095 to 0.519, which indicates better distribution. Consequently, no single compound can cross the BBB. Furthermore, the inhibitory or substrate response of the cytochrome P450 enzymes was used to confirm the metabolic pathways of the substances that were under investigation (CYPs). This enzyme is crucial to the oxidation process and helps ease the removal of foreign organic molecules like medicines. It also serves a crucial function in the production of energy in the cell. Ligands 04, 06 and 07 found to be actively inhibited the CYP450 1A2 Inhibitor, while no compounds were found to be CYP450 2C9 Substrate. In excretion prediction, the rate of total clearance is about 0.066 ml/min/kg to -6.618 ml/min/kg, which indicates a better clearance rate than clearance rate or negative score. The results of the toxicity tests, which included the AMES toxicity test and the hepatotoxicity test, suggest that the anticipated chemicals are safe, excluding 04, 06, and 07. On the other hand, the maximum tolerated dose in humans was in the range of 0.295–2.193 mg/kg/day.

**Table 5:** ADME and Toxicity prediction.

ADME and Toxicity prediction												
S/N	Absorption			Distribution		Metabolism		Excretion		Toxicity		
	Water solubility Log S	Human Intestinal Absorption (%)	Caco-2 Permeability+/-	VDss (human)	BBB Permeability+/-	CYP450 1A2 Inhibitor	CYP450 2C9 Substrate	Total Clearance (ml/min/kg)	Renal OCT2 substrate	AMES toxicity	Max. tolerated dose (human) mg/kg/day	Hepatotoxicity
L01	-0.252	61.19	+	-0.095	–	No	No	0.585	No	No	2.193	No
L02	-0.152	15.79	–	0.519	–	No	No	1.481	No	No	1.375	No
L03	-1.226	3.49	–	-0.156	–	No	No	1.642	No	No	0.396	No
L04	-2.892	100	–	0.008	–	Yes	No	6.918	No	Yes	0.438	No
L05	-1.177	0.00	–	-0.201	–	No	No	1.564	No	No	0.523	No
L06	-3.543	65.97	+	0.13	–	Yes	No	0.455	No	Yes	0.904	No
L07	-2.892	83.25	+	0.011	–	Yes	No	35.56	No	Yes	0.438	No
L08	-2.890	39.94	–	0.026	–	No	No	0.131	No	No	0.438	No
L09	-2.881	11.41	–	-0.265	–	No	No	0.066	No	No	0.295	No

“+ “ meaning Present/Positive & “-“ meaning Absent/Negative.

## Conclusion

Kaempferol-O-rhamnoside derivatives (L01-L09) have been studied for their potential as agonists to suppress Mpox and MARV infections; these compounds have been modeled using computational tools using DFT method, as well as molecular docking, molecular dynamics simulation, and in silico study.

In addition, investigations are conducted on pharmacokinetics, ADMET, drug-likeness, HOMO/LUMO gap, molecular electrostatic potential, and PASS prediction in an effort to offer them with an effective antiviral drug against Mpox and MARV infections. With beginning, the PASS prediction investigation outcome obtained at 0.379< Pa<0.712 for anti-viral (Influenza), 0.423< Pa<0.655 for anti-bacterial, 0.632< Pa<0.755 for antifungal, and 0.214< Pa<0.391 for anti-parasitic, so it can be said that they have a high possibility against viral pathogens. Next, molecular docking has been described as the most important part of this investigation, and it has achieved an acceptable docking score, which was even higher than the FDA-approved anti-viral Cidofovir drug. In accumulation, there are formed the hydrogen bonds after docking that conveys the more stability of protein as inhibitor. All of designed drugs showed the negative value of BBB permeability, CYP4501A2 inhibitor, CYP4502C9 substrate and Renal OCT2 substrate even they have no AMES toxicity. In case of quantum calculation, the energy gap is about 9.00 for 1^st^ four ligands (L01-L04) which is more reactive, and it is slightly lower for next class that is about 7.0 or below. However, the softness of L01 –L04 is about 0.21 where the softness for other is about 0.25 to 0.27. However, the 2^nd^ series of ligand (L05-L09) after joining is more active in case of docking but chemical more stable where L07, L08 and L09 show higher binding score. So, it can be said that with increasing the chain length, the binding capacity increases.

## Data availability statement

The original contributions presented in the study are included in the article/supplementary material. Further inquiries can be directed to the corresponding authors.

## Author contributions

AK, NM- Conceptualization, study design, writing, data acquisition. MA- Doing the simulation, DFT calculation and data analysis. AC, SM, AG, UC, SA, AK, AAla- Writing and data acquisition. AD, RS, AG, AAle and K-TC – Review, editing, and supervision. All authors contributed to the article and approved the submitted version.
